# Risk factors for loneliness among older informal caregivers in regions of Finland and Sweden: a longitudinal study

**DOI:** 10.1177/14034948241308029

**Published:** 2025-01-08

**Authors:** Sarah Åkerman, Fredrica Nyqvist, Mikael Nygård, Fredrik Snellman, Birgitta Olofsson

**Affiliations:** 1 Social Policy, Faculty of Education and Welfare Studies, Åbo Akademi University, Vaasa, Finland; 2 Social Work, Faculty of Social Sciences, Umeå University, Umeå, Sweden; 3 Nursing, Department of Nursing, Department of Surgery and Perioperative Sciences, Division of Orthopaedics, Umeå University, Umeå, Sweden

**Keywords:** Informal care, loneliness, longitudinal, social isolation, health, risk factors, old age, Nordic, Finland, Sweden

## Abstract

**Aims:**

This longitudinal study investigated the prevalence of and risk factors for loneliness among older new informal caregivers, long-term informal caregivers, former informal caregivers and non-caregivers in selected regions of Finland and Sweden over 5 years.

**Methods:**

A longitudinal sample of 5083 respondents from the Gerontological Regional Database (GERDA) survey data in 2016 and 2021 was used. Bivariate correlation tests and multivariate logistic regression analyses were performed.

**Results:**

Loneliness prevalence rates varied between 6% and 8% in 2016 and increased in all groups, although not significantly among new informal caregivers. In 2021, the prevalence of loneliness varied between 8% and 14%. Among the baseline variables, reporting loneliness, single/unmarried/bereaved civil status, infrequent contact with friends and neighbours, poor self-rated health, depressive symptoms, living in Finland and financial strain increased the likelihood of reporting loneliness in 2021. Among the change variables, being a long-term caregiver, a negative change in civil status, a reduced number of confidants, a negative change in self-rated health and depressive symptoms increased the likelihood of reporting loneliness.

**Conclusions:**

**Loneliness increased among all four groups of comparison. Being a long-term caregiver was a significant risk factor for reporting loneliness over time (odds ratio 2.00, 95% confidence interval 1.20–3.35), when controlling for several other social and health-related variables. To develop effective support, future research could address risk factors for loneliness among distinct groups of informal caregivers based on whether the care recipients have neurological or functional health limitations and examine the availability of support measures.**

## Background

Loneliness is considered a public health issue linked to morbidity and mortality [1, 2]. Loneliness can be defined as the subjective evaluation of a discrepancy between desired and actual resources [3], and is therefore a more complex phenomenon than merely the number of social contacts. Still, older adults are at particular risk of loneliness due to higher likelihood of a reduced social network caused by the declining health of one’s own or among age-matched peers [1]. Certain groups of older adults may be even more at risk, such as informal caregivers who are bound to the home [2] and/or provide care to someone with dementia [4]. Informal care refers here to care provided by family members and friends. In the United States and Europe, 17% of the entire population are informal caregivers, with prevalence rates dropping in older cohorts in the United States while increasing in Europe [5]. The present study aims to investigate loneliness in old age among four types of (non-) informal caregivers in selected regions in Finland and Sweden. Loneliness among informal caregivers has been found to be more prevalent in European regions with lower availability of formal care alternatives [6]. Finland and Sweden offer relatively advantageous settings for informal caregivers due to their generous and mainly tax-based welfare model [7, 8]. However, formal care for older adults has experienced retrenchment during the past decades in Finland and Sweden [7], tentatively contributing to heightened risks of loneliness among informal caregivers of older adults – especially in Finland where public spending on formal care for older adults is lower than in Sweden [8]. The present study focuses on older informal caregivers who typically provide care to a co-residing spouse with extensive care needs [9–11] and, as a result, may face greater risks of loneliness [12].

Some studies have found that informal caregiving is linked to increased levels of loneliness [2], while others have found positive social effects such as a deepened relationship with the care recipient and/or new social contacts related to the caregiver status [13]. A previous English longitudinal study on loneliness and informal care in old age found that, at baseline, a higher proportion of non-caregivers reported loneliness (11.8%) than informal caregivers (10.3%) [14], but that the prevalence of loneliness increased more among caregivers than among non-caregivers over the study period of 8 years. In a German study, Zwar et al. [15] studied the transition into the caregiver role and found that loneliness increased over time among male caregivers but not among female caregivers. The mixed results concerning the relationship between loneliness and informal care might be explained by the wide scope of informal care, which calls for studies in which informal caregivers are differentiated, for example, according to the stages of caregiving [16]. Consequently, this paper aspires to investigate the relationship between informal care and loneliness by categorising data from older adults [17] into four different types of (non-) informal caregivers. This categorisation is detailed in the data and method section.

## Aim

This paper will investigate the prevalence and associated risk factors for loneliness among older new informal caregivers, long-term caregivers, former caregivers and non-caregivers in selected regions in Finland and Sweden.

## Data and method

This paper uses data from the Gerontological Regional Data (GERDA) survey [17] in 2016 and 2021 conducted in the Bothnia region in Ostrobothnia and South Ostrobotnia, Finland and in Västerbotten, Sweden. The data collection in 2021 was extended and included also Åland in Southwest Finland. The multidisciplinary questionnaires, consisting of 92 questions and some theme modifications for each wave, can be found in Swedish and Finnish on https://www.gerdacenter.com/data-collections. In 2016, the questionnaires were sent out by post to every individual born in 1930, 1935, 1940, 1945 and 1950, except for in Vaasa and South Ostrobothnia in Finland where the questionnaires were sent to every second individual, and in the cities of Skellefteå and Umeå in Sweden, where every third individual was selected. The questionnaire was sent to 14,805 older adults and 9386 participated, resulting in a response rate of 63%. In 2021, the questionnaire was sent out to the same age cohorts with the additional age cohort of individuals born in 1955. The questionnaire was sent out to 23,169 individuals and 11,984 responded, resulting in a response rate of 52%.

This paper uses longitudinal individual-level data derived from the GERDA survey [17] waves in 2016 and 2021. The longitudinal data consists of 5083 respondents who participated in both 2016 and 2021. In the questionnaire, an informal caregiver was defined as ‘a person looking after a family member/. . ./ that due to illness, lowered functional capacity, or another reason needs help and support and therefore does not manage independently in everyday life’. Following the definition, the multiple response question ‘Who do you give informal care to?’ was offered. Participants were categorised as caregivers if they chose one of the two first response options (loved one in my household, loved one in another household, I do not give informal care to anyone). Four groups for comparison were selected for the analyses of the present study. The first group consisted of new caregivers (*N*=272), who identified as non-caregivers in 2016 and as informal caregivers in 2021. The second group represented long-term informal caregivers (*N*=243) and consisted of respondents who identified as informal caregivers in both 2016 and 2021. The third group consisted of former caregivers (*N*=396 respondents), who identified as informal caregivers in 2016 and as non-caregivers in 2021. The fourth group consisted of non-caregivers (*N*=2155) who were non-caregivers in both 2016 and 2021.

### Variables

#### Background variables

Background variables included age (‘66, 71, 76’ vs. ‘81, 86, 91’), male versus female gender, educational level (‘10 years or more’, ‘less than 10 years’) and financial strain. Financial strain was assessed with the question ‘Is it possible for you to make ends meet?’ and had four response options. The variable was dichotomised into ‘no financial strain’ and ‘financial strain’ (with some difficulty, with difficulty, with much difficulty).

#### Social variables

Loneliness was used as the outcome variable and was measured with the question ‘Do you suffer from loneliness?’ (no/yes). Marital status was dichotomised into two categories ‘married, co-habiting, partner’ and ‘widowed, single, unmarried’.

Two variables were used to measure contact frequency with family members and relatives, and with neighbours and friends, respectively. The original question ‘How often are you in contact with one/several of the following persons?’ had five response options. The variables were dichotomised into ‘frequent contact’ (several times a week) if the respondent had contact with at least one person in the category several times a week. ‘Infrequent contact’ (several times a month, a few times a year, never, the person does not exist) indicated contact less often than several times a week.

The number of confidants was assessed with the question, ‘Do you have a confidant with whom you can speak about anything that is sharing both concerns and joys?’ with multiple options of, for example, relatives, friends and staff. After calculating median split, the variable was dichotomised into ‘2 confidants or more’ and ‘0–1 confidants’.

#### Health variables

Self-rated health was assessed with the question: ‘In general, how would you say your health is?’. The variable was dichotomised into ‘good’ (excellent, very good, good) and ‘poor’ (fair, poor). Depressive symptoms were assessed with the self-reporting question, ‘Do you feel depressed?’ (no/yes).

#### Country

Country was included as an explanatory variable (Sweden/Finland).

#### Change variables

Change variables were created by combining variables from 2016 and 2021. The 2016 scores were subtracted from the 2021 scores. The change scores were dichotomised into no change/positive change versus negative change.

#### Analyses

All variables’ distribution (%) was calculated according to the four groups of comparison (Table I). The McNemar test was used to study significant differences in the levels of loneliness between the two time points of study in each group of caregivers, respectively. Contingency tables with Pearson’s chi-square tests were used to analyse the bivariate associations between the explanatory variables at baseline and loneliness in 2021 among the different groups of caregivers.

**Table I.** Distribution of variables (%/*N*) in 2016 and 2021 and bivariate association by Pearson chi-square between baseline characteristics and loneliness in 2021 among new caregivers, long-term caregivers, former caregivers and non-caregivers.

	New caregivers (*n*=272)			Long-term caregivers (*n*=243)			Former caregivers (*n*=396)			Non-caregivers (*n*=2155)		
	2016	2021	*P* value	2016	2021	*P* value	2016	2021	*P* value	2016	2021	*P* value
Age												
66, 71, 76	86.4 (235)	63.2 (172)	ns	86.4 (210)	66.7 (162)	ns	68.4 (271)	85.4 (338)	*	69.9 (1506)	86.9 (1873)	***
81, 86, 91	13.6 (37)	36.8 (100)		13.6 (33)	33.3 (81)		31.6 (125)	14.6 (58)		30.1 (649)	13.1 (282)	
Gender												
Male	48.5 (132)		ns	43.2 (105)		ns	47.5 (188)		ns	50.8 (1095)		***
Female	51.5 (140)			56.8 (138)			52.5 (208)			49.2 (1059)		
Educational level												
Higher	62.6 (169)		ns	67.8 (164)		ns	66.9 (263)		ns	63.6 (1358)		ns
Lower	37.4 (101)			32.2 (78)			33.1 (130)			36.4 (776)		
Financial strain												
No	66.0 (175)	69.5 (185)	ns	71.4 (170)	66.4 (160)	*	66.4 (259)	63.9 (248)	*	68.4 (1450)	66.6 (1420)	***
Yes	34.0 (90)	30.5 (81)		28.6 (68)	33.6 (81)		33.6 (131)	36.1 (140)		31.6 (671)	33.4 (711)	
*Social*												
Loneliness												
No	93.2 (247)	91.8 (245)	ns	94.1 (225)	88.5 (208)	*	91.6 (348)	86.3 (334)	*	91.9 (1931)	90.3 (1989)	*
Yes	6.8 (18)	8.2 (22)		5.9 (14)	11.5 (27)		8.4 (32)	13.7 (53)		8.1 (171)	9.7 (204)	
Civil status												
Married, co-habiting, partner	90.4 (245)	91.4 (245)	ns	90.1 (219)	85.7 (204)		85.3 (337)	64.8 (254)	*	74.3 (1595)	69.0 (1472)	***
Widowed, divorced, single	9.6 (26)	8.6 (23)		9.9 (24)	14.3 (34)		14.7 (58)	35.2 (138)		25.7 (551)	31.0 (660)	
Contact with family members												
Frequent	89.0 (242)	89.7 (244)	ns	89.7 (218)	88.5 (215)	ns	92.2 (365)	79.8 (316)	ns	83.8 (1806)	81.1 (1748)	***
Infrequent	11.0 (30)	10.3 (28)		10.3 (25)	11.5 (28)		7.8 (31)	20.2 (80)		16.2 (349)	18.9 (407)	
Contact with friends and neighbours												
Frequent	40.1 (109	35.3 (96)	ns	42.4 (103)	42.4 (103)	ns	43.4 (172)	42.9 (170)	ns	42.5 (916)	38.7 (835)	*
Infrequent	59.9 (163)	64.7 (176)		57.6 (140)	57.6 (140)		56.6 (224)	57.1 (226)	ns	57.5 (1239)	61.3 (1320)	
Number of confidants												
2 or more	73.2 (199)	76.5 (208)	ns	72.0 (175)	71.2 (173)	ns	68.4 (271)	72.0 (285)	ns	66.8 (1440)	68.6 (1479)	ns
0–1	26.8 (73)	23.5 (64)		28.0 (68)	28.8 (70)		31.6 (125)	28.0 (111)		33.2 (715)	31.4 (676)	
Self-rated health												
Poor	25.9 (70)	40.1 (108)	ns	32.8 (79)	34.6 (83)	**	28.9 (114)	36.0 (141)	***	27.1 (582)	32.4 (692)	***
Good	74.1 (200)	59.9 (161)		67.2 (162)	65.4 (157)		71.1 (280)	64.0 (251)		72.9 (1563)	67.6 (1441)	
Depressive symptoms												
Yes	93.6 (248)	89.9 (238)	ns	94.0 (219)	90.9 (211)	***	94.5 (360)	91.0 (353)	***	93.2 (1955)	91.5 (1918)	***
No	6.4 (17)	10.2 (27)		6.0 (14)	9.1 (21)		5.5 (21)	9.0 (35)		6.8 (143)	8.5 (178)	
Country												
Sweden	60.7 (165)		ns	56.4 (137)		ns	50.3 (199)		ns	56.4 (1215)		ns
Finland	39.3 (107)			43.6 (106)			49.7 (197)			43.6 (940)		

* *P*<0.05, **P<0.01, ***P<0.001.

For loneliness, the significance levels are based on results from the McNemar Test for paired data.

Logistic regressions were conducted by calculating odds ratios (ORs) with 95% confidence intervals (CIs) for the likelihood of reporting loneliness in 2021 by informal caregiver status, social, health, background, country and change variables (Table II). Six models were estimated, and the variables were entered stepwise in the following sequence: (a) informal caregiver status; (b) background variables; (c) social variables; (d) health variables; (e) country; and (f) change variables. Multicollinearity statistics were run, and variance influence factors ranged between 1 and 1.5. All analyses were performed using the statistical program IBM SPSS Statistics 29.

**Table II.** Odds ratios (ORs) and 95% confidence intervals (CIs) for the probability of reporting loneliness in 2021.

	Model 1	Model 2	Model 3	Model 4	Model 5	Model 6
Informal caregiver status 2016–2021						
Non-caregiver	1	1	1	1	1	1
Long-term caregiver	1.21 (0.79–1.85)	1.24 (0.80–1.91)	1.49 (0.94–2.36)	1.49 (0.93–2.38)	1.49 (0.93–2.38)	2.00 (1.20–3.35)**
New informal caregiver	0.84 (0.53–1.32)	0.84 (0.53–1.35)	0.94 (0.57–1.55)	0.98 (0.59–1.62)	0.98 (0.60–1.63)	1.06 (0.60–1.88)
Former caregiver	1.48 (1.07–2.04)*	1.46 (1.05–2.03)*	1.54 (1.07–2.21)*	1.54 (1.06–2.23)*	1.53 (1.06–2.22)*	1.23 (0.80–1.88)
Background variables (2016)						
Age 66 71 76		1	1	1	1	1
Age 81 86 91		2.41 (1.79–3.26)***	2.11 (1.52–2.93)***	1.88 (1.34–2.65)***	1.88 (1.33–2.64)***	1.39 (0.95–2.04)
Male gender		1	1	1	1	1
Female gender		1.60 (1.25–2.05)***	1.35 (1.03–1.77)*	1.26 (0.96–1.66)	1.26 (0.95–1.66)	1.21 (0.89–1.66)
Upper secondary education		1	1	1	1	1
Lower secondary education		1.03 (0.80–1.33)	1.05 (0.80–1.37)	0.98 (0.74–1.29)	0.98 (0.74–1.30)	0.90 (0.66–1.22)
No financial strain		1	1	1	1	1
Financial strain		1.90 (1.49–2.43)***	1.57 (1.20–2.04)***	1.42 (1.08–1.86)*	1.42 (1.08–1.86)*	1.48 (1.08–2.04)*
Social variables (2016)						
No loneliness			1	1	1	1
Loneliness			5.91 (4.27–8.18)***	4.27 (2.93–6.22)***	4.28 (2.94–6.24)***	3.97 (2.60–6.04)***
Civil status: married, co-habiting, partner			1	1	1	1
Widow, single, unmarried			1.42 (1.03–1.96)*	1.49 (1.07–2.07)*	1.51 (1.08–2.10)*	2.47 (1.67–3.67)***
Frequent contact with family members			1	1	1	1
Infrequent contact with family members			1.33 (0.94–1.90)	1.30 (0.91–1.85)	1.27 (0.88–1.81)	1.07 (0.71–1.63)
Frequent contact with friends and neighbours			1	1	1	1
Infrequent contact with friends and neighbours			1.31 (1.00–1.72)	1.28 (0.97–1.69(	1.27 (0.96–1.68)	1.70 (1.12–2.48)*
Number of confidants: more than 1			1	1	1	1
Number of confidants: 0–1			1.20 (0.91–1.59)	1.16 (0.87–1.53)	1.17 (0.88–1.56)	1.32 (0.94–1.85)
Health variables (2016)						
Good self-rated health				1	1	1
Poor self-rated health				1.97 (1.50–2.60)***	1.97 (1.49–2.59)***	2.00 (1.44–2.80)***
No depressive symptoms				1	1	1
Depressive symptoms				1.83 (1.20–2.80)**	1.83 (1.20–2.81)**	2.87 (1.80–4.56)***
Country (2016)						
Sweden					1	1
Finland					1.11 (0.85–1.46)	1.46 (1.07–1.98)*
Changes in background, social, and health variables (2016–2021)						
Financial strain: negative change						1.27 (0.80–1.99)
Civil status: negative change						4.11 (2.58–6.55)***
Contact frequency with family members: negative change						1.47 (0.93–2.33)
Contact frequency with friends and neighbours: negative change						1.55 (0.95–2.54)
Number of confidants: negative change						1.70 (1.11–2.60)*
Self-rated health: negative change						1.71 (1.10–2.66)*
Perceived depressive symptoms: negative change						12.21 (8.14–18.33)***
Log likelihood	1967.91	1857.12	1658.27	1597.12	1596.53	1298.85
Cox and Snell R	0.002	0.027	0.072	0.083	0.083	0.151
Nagelkerke R	0.005	0.056	0.149	0.172	0.172	0.318

* *P*<0.05, **P<0.01, ***P<0.001.

#### Ethical considerations

The study follows the guidelines of the Finnish advisory board on research integrity [18]. The data collection was approved by the regional ethical review board in Umeå, Sweden in 2016 (2016-367-32) and 2021 (2021-04965, 05-084Ö).

## Results

Loneliness increased significantly among all groups except for new informal caregivers (Table I). Among new caregivers, loneliness prevalence rates increased from 6.8% to 8.2% between 2016 and 2021. Among long-term caregivers, it increased from 5.9% to 11.5%. Among former caregivers, it increased from 8.4% to 13.7%. Among non-caregivers, loneliness prevalence rates increased from 8.1% to 9.7%.

In model 1 in the logistic regression (Table II), being a former caregiver increased the likelihood of reporting loneliness at follow-up in 2021 (OR 1.48, 95% CI 1.07–2.04). In model 2, when background variables were entered, higher age (OR 2.41, 95% CI 1.79–3.26), female gender (OR 1.60, 95% CI 1.25–2.05) and financial strain (OR 1.90, 95% CI 1.49–2.43) increased the likelihood of reporting loneliness. In model 3, when social variables were entered, reporting loneliness at baseline (OR 5.91, 95% CI 4.27–8.18), and single, unmarried, or widowed civil status (OR 1.42, 95% CI 1.03–1.96) and infrequent contact with friends and neighbours (OR 1.35, 95% CI 1.03–1.77) increased the likelihood of reporting loneliness at follow-up. In model 4, when health variables were entered, the relationship between gender and loneliness disappeared. Poor self-rated health (OR 2.14, 95% CI 1.63–2.82) and depressive symptoms (OR 1.84, 95% CI 1.20–2.80) increased the likelihood of reporting loneliness at follow-up. In model 5, country was added but it was not significant. In model 6, when change variables were entered, being a former caregiver ceased to predict loneliness, while being a long-term caregiver (OR 2.00, 95% CI 1.20–3.35) became significant. Infrequent contact with friends and neighbours increased the likelihood of reporting loneliness (OR 1.70, 95% CI 1.12–2.48). Age ceased to be significant. Living in Finland increased the likelihood of reporting loneliness (OR 1.46, 95% CI 1.07–1.98). Among the change variables, a negative change in civil status (OR 4.11, 95% CI 2.58–6.55), number of confidants (OR 1.70, 95% CI 1.11–2.60), self-rated health (OR 1.71, 95% CI 1.10–2.66) and depressive symptoms (OR 12.21, 95% CI 8.14–18.33) increased the likelihood of reporting loneliness at follow-up.

## Discussion

This paper aimed to investigate the prevalence and associated risk factors for loneliness among new informal caregivers, long-term caregivers, former caregivers and non-caregivers in selected regions in Finland and Sweden. Loneliness prevalence rates increased in all groups between the two timepoints, although not significantly among new informal caregivers. At follow-up in 2021, the prevalence of loneliness varied between 8.2% and 13.7%. Apart from informal caregiver status, the results of the final multivariate model showed that baseline variables such as widowed/single/unmarried civil status, infrequent contact with friends and neighbours, poor-self-rated health, depressive symptoms, living in Finland, experiencing financial strain, negative change in the number of confidants, negative changes in self-rated health and perceived depressive symptoms increased the likelihood of reporting loneliness in 2021.

Among new informal caregivers, the proportion of respondents reporting frequent contact with friends and neighbours decreased among new informal caregivers (from 40.1% to 35.3%) over the span of 5 years, while there was no change in the proportion of caregivers having frequent contact with family members. Previous studies have pointed out the ambiguous role of social resources among informal caregivers, in which informal care might lead to social isolation, in terms of the absence of social contacts or to a deepened or expanded social network [2, 13]. Zwar et al. [15] found that transitioning into the informal caregiver role was associated with an increase in the social network. Male caregivers were at risk of reporting loneliness at follow-up, while this did not apply to female caregivers. In the present study, gender was not associated with loneliness in the final multivariate models. Out of the caregiver types, being a long-term caregiver was the only significant risk factor for loneliness in the final model. A tentative explanation could be that, at critical phases such as when transitioning in or out of the caregiver role, practical challenges overrule feelings of loneliness – while during long periods of informal caregiving, loneliness becomes more prevalent.

Former caregivers reported the highest prevalence of loneliness at follow-up (13.7%). Previous studies have disclosed that they may struggle to find an appropriate social context post-caregiving [19, 20]. Being a former caregiver was associated with loneliness in the first models in the multivariate analysis, but the relationship disappeared when controlling for a negative change in civil status, tentatively indicating that widowhood is a stronger risk factor for loneliness than ceasing to be a caregiver. Among non-caregivers, loneliness prevalence rates increased slightly but significantly from 8.1% to 9.7%. One possible contributing factor to the increased levels of loneliness in all four groups of comparison could be the COVID-19 pandemic that coincided with the second wave of the survey. Some studies have found that both older adults in general [21, 22] and informal caregivers [23] reported increased levels of loneliness and/or a negative impact on their social network during the pandemic. A longitudinal population-based study conducted in England [24] found that loneliness levels decreased among non-caregivers during the pandemic (from 7.5% to 7.1%) and that loneliness increased only slightly among caregivers (from 8.0% to 8.5%). The notable increase in loneliness observed in the present study might be explained by the study population being older and thus more likely to be exposed to social and health related risk factors for loneliness [1, 22, 25].

Reporting loneliness at baseline was a strong predictor for reporting loneliness at follow-up. In addition, widowed/single/unmarried civil status, infrequent contact with friends and neighbours, poor self-rated health, depressive symptoms, experiencing financial strain, negative change in the number of confidants, negative change in self-rated health and perceived depressive symptoms increased the likelihood of reporting loneliness in 2021. These findings are in line with previous research on risk factors for loneliness in old age [1, 25]. In addition, living in Finland increased the likelihood of reporting loneliness in comparison to living in Sweden, and this finding is corroborated by a study on loneliness in old age using repeated cross-sectional GERDA data [22] albeit contradictory to a pre-pandemic longitudinal study [25]. As discussed by the authors [22], the more recent results might be explained by the pandemic and stricter social regulations in Finland. Another possible explanation for the results of the present study is that the two countries have adapted slightly different responses to the ageing population with, for example, a stronger emphasis on informal care in Finland than in Sweden [7, 26]. A study comparing recent reforms in social and health care for older adults found that, although the Finnish system holds advantages such as better care coordination, the Swedish system is more generous in terms of public spending [27]. Given that older informal caregivers typically provide care to older adults, this might translate into Finnish informal caregivers facing a greater burden than their Swedish peers. Simultaneously, the support for informal caregivers is more standardised in Finland, with legislated rights to economic allowances, insurance, training and health examinations [28, 29]. To assist policymakers in developing support to alleviate loneliness, future studies could further explore the links between loneliness and support measures for informal caregivers and/or care recipients. In addition, future research should distinguish informal caregivers according to, for example, whether the care recipient has neurological or functional limitations [4] and potential links to available support measures to understand loneliness further among informal caregivers and develop tailored support.

## Strengths and limitations

In any longitudinal survey targeting older adults, there are risks of sample selection bias due to healthier respondents being more likely to fill out the questionnaire. Similarly, strained informal caregivers might be less likely to participate in surveys as compared to non-strained caregivers. The sample used for the analyses consisted of 3066 individuals, due to 2016 missing responses on the variable assessing informal caregiver status. The low response rate might tentatively be explained by non-caregivers skipping the question about informal caregiver status. The proportion of informal caregivers in the GERDA survey in 2016 was similar to that of previous surveys mapping informal care in old age in Finland and Sweden [26, 29, 30]. In addition, the overall response rate at baseline (63.0%) and follow-up (55.3%) was relatively high further adding to the representativeness of the data.

The second wave of the survey took place during the aftermath of the COVID-19 pandemic which may have biased the results regarding social contacts and loneliness. Additional waves and analyses are needed to establish further the links between loneliness and informal care in this region.

The variable assessing loneliness only included the response options of ‘yes’ and ‘no’, although Likert scales are commonly used. Furthermore, the variable used in this study does not discriminate between social and emotional loneliness [30]. Information is also missing about the caregiver status between the two timepoints of study, which affects the validity of the results. This kind of categorisation has, however, been used previously [15].

## Conclusions

Our longitudinal study adds insights into a Nordic regional perspective on loneliness among older new informal caregivers, long-term caregivers, former caregivers and non-caregivers. Loneliness increased among all four groups across the study period of 5 years, although not significantly among new informal caregivers. Being a long-term caregiver was a significant risk factor for reporting loneliness at follow-up, after controlling for various social and health-related variables. To develop effective support, future research should address risk factors for loneliness among distinct groups of informal caregivers based on whether the care recipients have neurological or functional health limitations and examine the availability of support measures.

## References

[1] Dahlberg L McKee KJ Frank A , et al. A systematic review of longitudinal risk factors for loneliness in older adults. Aging Ment Health 2022;26:225–249. doi:10.1080/13607863.2021.1876638 33563024

[2] Hajek A Kretzler B König HH. Informal caregiving, loneliness and social isolation: a systematic review. Int J Environ Res Public Health 2021;18:12101. doi:10.3390/ijerph182212101 34831857 PMC8618455

[3] Perlman D Peplau LA. Toward a Social Psychology of Loneliness. In: Gilmour R Duck S (eds) Personal Relationships: Relationships in Disorder. London: Academic Press, 1981, pp. 31–756.

[4] Saadi JP Carr E Fleischmann M , et al. The role of loneliness in the development of depressive symptoms among partnered dementia caregivers: evidence from the English Longitudinal Study of Aging. Eur Psychiatry 2021;64:e28. doi:10.1192/j.eurpsy.2021.204 PMC8080187 33766187

[5] Tur-Sinai A Teti A Rommel A , et al. How many older informal caregivers are there in Europe? Comparison of estimates of their prevalence from three European surveys. Int J Environ Res Public Health 2020;17:9531. doi:10.3390/ijerph172495316 33352669 PMC7767284

[6] Wagner M Brandt M. Long-term care provision and the well-being of spousal caregivers: an analysis of 138 European regions. J Gerontol B Psychol Sci Soc Sci 2018;73:e24–e34. doi:10.1093/geronb/gbx133 PMC6018933 29237034

[7] Rostgaard T Jacobsen F Kröger T , et al. Revisiting the Nordic long-term care model for older people-still equal? Eur J Ageing 2022;16;19:1641. doi: 10.1007/s10433-022-00712-3 PMC9729475 36506690

[8] Verbakel E. How to understand informal caregiving patterns in Europe? The role of formal long-term care provisions and family care norms. Scand J Public Health 2018;46:436–447. doi:10.1177/1403494817726197 28823224 PMC5989248

[9] Bom J Bakx P Schut F , et al. The impact of informal caregiving for older adults on the health of various types of caregivers: a systematic review. Gerontologist 2019;59:e629–e642. doi:10.1093/geront/gny13710 PMC6850889 30395200

[10] Adelman RD Tmanova LL Delgado D , et al. Caregiver burden: a clinical review. JAMA 2014;311:1052–1060. doi:10.1001/jama.2014.304 24618967

[11] Schulz R Beach SR Czaja SJ , et al. Family caregiving for older adults. Annu Rev Psychol 2020;71:635–659. doi:10.1146/annurev-psych-010419-050754 31905111 PMC7291827

[12] Li L Wister AV Mitchell B. Social isolation among spousal and adult–child caregivers: findings from the Canadian Longitudinal Study on Aging. J Gerontol B Psychol Sci Soc Sci 2021;76:1415–1429. doi:10.1093/geronb/gbaa197 33170276 PMC8363033

[13] Pysklywec A Plante M Auger C , et al. The positive effects of caring for family carers of older adults: a scoping review. Int J Care Caring 2020;4:349–375. doi:10.1332/239788220x15925902138734

[14] Smith T Saunders A Heard J. Trajectory of psychosocial measures amongst informal caregivers: case-controlled study of 1375 informal caregivers from the English Longitudinal Study of Ageing. Geriatrics 2020;5:26. doi:10.3390/geriatrics5020026 32349243 PMC7345989

[15] Zwar L König HH Hajek A. Psychosocial consequences of transitioning into informal caregiving in male and female caregivers: findings from a population-based panel study. Soc Sci Med 2020;264:113281. doi:10.1016/j.socscimed.2020.113281 32829215

[16] Rafnsson SB . Loneliness and social isolation among older informal caregivers: a review of the evidence from longitudinal investigations. In: Hajek A Riedel-Heller SG König HH (eds) Loneliness and Social Isolation in Old Age: Correlates and Implications (1st ed.), Routledge. 10.4324/9781003289012

[17] Åbo Akademi University. The GERDA survey. Åbo Akademi University, Social Sciences, 2021. http://urn.fi/urn:nbn:fi:att:e917092f-e9c5-4a7b-91b5-bb704e3daeb9

[18] Finnish Advisory Board on Research Integrity. Guidelines of the Finnish Advisory Board on Research Integrity, 2023. https://tenk.fi/sites/default/files/2023-11/RI_Guidelines_2023.pdf

[19] Åkerman S Nyqvist F Coll-Planas L , et al. The expert caregiver intervention targeting former caregivers in Finland: a co-design and feasibility study using mixed methods. Int J Environ Res Public Health 2021;18:10133. 10.3390/ijerph181910133 34639437 PMC8508176

[20] Mora-Lopez G Berenguer-Poblet M Berbis-Morelló C , et al. New life transition of former caregivers: positive mental health approach. Front Psychol 2022;13:854108. doi:10.3389/fpsyg.2022.854108 35444601 PMC9013805

[21] Su Y Rao W Li M , et al. Prevalence of loneliness and social isolation among older adults during the COVID-19 pandemic: a systematic review and meta-analysis. Int Psychogeriatr. 2023;35:229–241. doi:10.1017/S1041610222000199 35357280

[22] Nyqvist F Nilsson I Näsman M , et al. The association between leisure engagement and loneliness before and during the COVID-19 pandemic: a Nordic population-based study. Scand J Public Health 2023;51:744–753. doi:10.1177/14034948231171964 37165572 PMC10183342

[23] Beach SR Schultz R Donovan H , et al. Family caregiving during the COVID-19 pandemic. Gerontologist 2021;61:650–660. doi:10.1093/geront/gnab049 33847355 PMC8083337

[24] Gallagher S Wetherell MA. Risk of depression in family caregivers: unintended consequence of COVID-19. BJPsych Open 2020;6:e119. doi:10.1192/bjo.2020.99 PMC7550870 33040759

[25] Nyqvist F Näsman M Hemberg J , et al. Risk factors for loneliness among older people in a Nordic regional context – a longitudinal study. Ageing Society 2023;43:2372–2393. doi:10.1017/S0144686X21001707

[26] Åkerman S Nyqvist F Nygård M. A cross-sectional study on the associations between economic, social, and political resources and subjective caregiver burden among older spousal caregivers in two Nordic regions. Nurs Rep 2023;13:365–377. doi:10.3390/nursrep1301003427 36976686 PMC10053522

[27] Tynkkynen LK Pulkki J Tervonen-Gonçalves L , et al. Health system reforms and the needs of the ageing population – an analysis of recent policy paths and reform trends in Finland and Sweden. Eur J Ageing 2022;19:221–232. doi:10.1007/s10433-022-00699-x 35465210 PMC9012246

[28] Ministry of Social Affairs. Next of Kin Who Provide or Support Close Older Adults. Foundation for a National Strategy, 2020. https://www.socialstyrelsen.se/globalassets/sharepoint-dokument/artikelkatalog/ovrigt/2021-6-7464.pdf29

[29] Ministry of Social Affairs and Health. The development of informal care and family care during 2015–2018. Conclusions and recommendations for further measures, 2019. http://urn.fi/URN:ISBN:978-952-00-4022-2

[30] Weiss RS. Loneliness: the Experience of Emotional and Social Isolation. Cambridge, MA: MIT Press, 1973.

